# Improved Activity of a Thermophilic Cellulase, Cel5A, from *Thermotoga maritima* on Ionic Liquid Pretreated Switchgrass

**DOI:** 10.1371/journal.pone.0079725

**Published:** 2013-11-14

**Authors:** Zhiwei Chen, Jose H. Pereira, Hanbin Liu, Huu M. Tran, Nathan S. Y. Hsu, Dean Dibble, Seema Singh, Paul D. Adams, Rajat Sapra, Masood Z. Hadi, Blake A. Simmons, Kenneth L. Sale

**Affiliations:** 1 Deconstruction Division, Joint BioEnergy Institute, Emeryville, California, United States of America; 2 Biomass Science and Conversion Technology, Sandia National Laboratories, Livermore, California, United States of America; 3 Physical Biosciences Division, Lawrence Berkeley National Laboratory, Berkeley, California, United States of America; 4 Department of Bioengineering, University of California Los Angeles, Los Angeles, California, United States of America; 5 Department of Bioengineering, University of California, Berkeley, California, United States of America; University of Nottingham, United Kingdom

## Abstract

Ionic liquid pretreatment of biomass has been shown to greatly reduce the recalcitrance of lignocellulosic biomass, resulting in improved sugar yields after enzymatic saccharification. However, even under these improved saccharification conditions the cost of enzymes still represents a significant proportion of the total cost of producing sugars and ultimately fuels from lignocellulosic biomass. Much of the high cost of enzymes is due to the low catalytic efficiency and stability of lignocellulolytic enzymes, especially cellulases, under conditions that include high temperatures and the presence of residual pretreatment chemicals, such as acids, organic solvents, bases, or ionic liquids. Improving the efficiency of the saccharification process on ionic liquid pretreated biomass will facilitate reduced enzyme loading and cost. Thermophilic cellulases have been shown to be stable and active in ionic liquids but their activity is typically at lower levels. Cel5A_*Tma*, a thermophilic endoglucanase from Thermotoga maritima, is highly active on cellulosic substrates and is stable in ionic liquid environments. Here, our motivation was to engineer mutants of Cel5A_*Tma* with higher activity on 1-ethyl-3-methylimidazolium acetate ([C_2_mim][OAc]) pretreated biomass. We developed a robotic platform to screen a random mutagenesis library of Cel5A_*Tma*. Twelve mutants with 25–42% improvement in specific activity on carboxymethyl cellulose and up to 30% improvement on ionic-liquid pretreated switchgrass were successfully isolated and characterized from a library of twenty thousand variants. Interestingly, most of the mutations in the improved variants are located distally to the active site on the protein surface and are not directly involved with substrate binding.

## Introduction

Uncertainties in petroleum supplies [1], concerns about climate change [2], and environmental pollution [3] have generated renewed interest in producing sustainable biofuels and bio-derived chemicals from lignocellulosic biomass [4], [5]. Cellulose is the most abundant renewable carbon source on the planet and therefore has the potential to provide a large quantity of the worldwide demand for transportation fuels and commodity chemicals [6]; the solar energy stored in biomass is estimated to be around two thousand Exajoule (10^18^ J) per year, which is ten fold larger than the energy content of the world’s annual petroleum production [7]. However, the current utilization of biomass to produce energy represents only about 2 % of the annual production of biomass in the world [8].

A major obstacle to the conversion of lignocellulosic biomass to fuels is that the highly complex lignocellulosic matrix consisting of cellulose, hemicellulose and lignin is highly recalcitrant to degradation into its individual components. Breaking this recalcitrance requires pretreatment using methods such as dilute acids [9], ammonia fiber expansion (AFEX) [10], hot water treatment [11] and steam explosion [12], before enzymes can efficiently access the cellulose and hemicelluloses and generate fermentable sugars. Pretreatment with ionic liquids, such as 1-ethyl-3-methylimidazolium acetate ([C_2_mim][OAc]), has recently been demonstrated as an extremely effective method of liberating cellulose from lignocellulosic biomass in a form that is easily converted to glucose, requiring lower enzyme loading and shorter hydrolysis times to reach 90 % yields [13].

Cellulose is a polysaccharide consisting of a linear chain of several hundred to over ten thousand β(1→4) linked D-glucose units, comprises 35–50 % of lignocellulosic biomass and is the major biomass component for hydrolysis [14], [15]. Breaking cellulose down to glucose, the final product of enzymatic hydrolysis, is carried out by the actions of enzymes known as cellulases, which include endoglucanases (EC 3.2.1.4), cellobiohydrolases (EC 3.2.1.91) and beta-glucosidases (EC 3.2.1.21) [16]. Cellulases are one of the most important biocatalysts in cellulosic fuels production [17], and currently are a major contributor to the overall cost of a gallon of biofuel [18]. While specific (hyper-) thermophilic cellulases from bacteria and archaea have been shown to be more stable and active in ionic liquids (ILs) than their mesophilic eukaryotic counterparts [19], [20], their activity in these environments is typically diminished, and it is desired to improve their activity under these conditions using enzyme engineering.

Cellulases, especially thermophilic cellulases, have proven to be difficult targets for improving activity and catalytic efficiency either by rational design or directed evolution and few successes have been reported [15], [16], [21]. Most of these studies were focused on improving thermostability [22]–[24], and only one was focused on improving catalytic efficiency [25]. Microbial and enzyme based screening methods have been developed for most of these cases [25], [26]. Cel5A from Thermotoga maritima (Cel5A_*Tma*) MSB8 (NCBI reference sequence NP_229549.1) [27]–[32], one of the most studied thermophilic endoglucanases, has promising characteristics, such as high activity, thermostability, multi-specificity on different polysaccharides [29], and tolerance to specific ionic liquids [19] that make it a good candidate for use in cellulase cocktails designed to operate at biorefinery relevant conditions, and we have selected this enzyme to be the focus of a directed evolution approach to improve its performance.

Directed evolution of enzymes relies on using a highly sensitive assay to screen for a relatively small number of enhanced variants within a library containing possibly tens of thousands of null mutants [33]. Although several automated enzymatic assays have been developed for cellulase screening, most of them are complicated, not fully automatic and limited to reactions at low temperature (room temperature to 50°C) [34]–[36]. To identify the evolved thermophilic endoglucanase, Cel5A_*Tma* with improved activity, we developed a high throughput cellulase activity assay at high temperature (70°C). Using this high-throughput screening platform, we screened a library of Cel5A_*Tma* in which mutations were inserted at random positions using error-prone PCR. Mutants were prescreened for improved activity on the soluble substrate, carboxymethyl cellulose (CMC). From a library of twenty thousand variants, twelve mutants with increased activity (25–42 %) were sequenced and confirmed for improved specific activity on CMC. The library of twelve mutants with enhanced specific activity on CMC was further screened for activity on [C_2_mim][OAc] pretreated switchgrass (ILSG); three of the twelve mutants also showed improvements on ILSG (13–30%). Structural analyses were used to analyze the effects of mutations in the improved Cel5A_*Tma* mutants. Intriguingly, most of the mutation sites are located on the molecular surface at positions distal to the active site.

## Materials and Methods

### Protein Expression and Purification

The pCDF2-*cel*5a_*Tma* construct containing the endoglucanase cel5a gene from Thermotoga maritima MSB8 [31] was used for protein expression and mutagenesis. BL21 (DE3) (EMD Biosciences) or Acella (EdgeBio) strains carrying the *cel*5a_*Tma* gene and mutants thereof were inoculated into LB autoinduction media with 100 µg/mL of streptomycin using Overnight Express Autoinduction System 1 and incubated at 30°C for 24 h. Cell pellets were then used either directly for protein purification or stored at -80°C. Proteins were extracted by Protein Extraction Buffer (1x BugBuster, 1 mg/mL of lysozyme, 1x Benzonase and 1x Protease Inhibitor Cocktail Set V EDTA-free), purified by Ni-NTA Spin Columns (Qiagen) and buffer-exchanged using Zeba Spin Desalting Columns (2 mL, 7 k MWCO, Pierce) pre-equilibrated with Storage Buffer (20 mM Tris-HCl and 50 mM NaCl, pH 7.20). The final purity of proteins was analyzed by SDS-PAGE (Novex 8-16 % Tris-Glycine Gel, Invitrogen) stained with Coomassie Blue R-250. Concentrations of the proteins were measured by bicinchoninic acid assay (BCA1 kit, Sigma) using bovine serum albumin as the standard and UV absorbance at 280 nm using the molar extinction coefficient of Cel5A_*Tma* (ε = 99,550 M^−1^·cm^−1^).

### Biomass Pretreatment

Cave-in-Rock switchgrass was harvested at the anthesis (R4) stage and contained 8.5 % (w/w) moisture as measured using an automatic moisture analyzer (Model HB 43-S, Mettler Toledo) utilizing a 10-min and 105-°C constant temperature program. Switchgrass (88.39 g, 8.49 % (w/w) moisture, 7.89 dry % (w/w)) was added to 924.36 g [C2mim][OAc] (>90% purity, BASF) at 27°C in a 1-l glass reaction flask equipped with an electronically controlled heating mantle, thermocouple probe, continuous nitrogen purge, condenser with distillate take off, and stirring system with a 76-mm turbine impeller and stirring torque monitor. The temperature of the slurry was ramped to 140°C and held for three hours with continuous stirring before cooling to 60°C.

The warm viscous solution was then mixed with 3,000 mL of boiling water in a plastic bucket, and the solution was homogenized in 500 mL aliquots with a laboratory blender (Model LB10G, Waring) at high speed for 20 s. The combined homogenized slurry was centrifuged (7000x g for 20 min, Avanti T-25, Beckman Coulter), and the recovered solid material was again washed and centrifuged in 7 stages with 3,000 mL of boiling water per stage. The combined slurry resulting from this wash process (2.6 % (w/w) solids, 55.9 % of initial SG solids) was then extracted under nitrogen in a large soxhlet extraction system (size H, glass thimble with frit base, porosity A (145–175 µm), appprox. 75 min per extraction cycle, Ace Glass) for 20 h with 95 % (v/v) ethanol and dried in a vacuum oven at 40°C. The resulting dry product contained approximately 0.15 % (w/w) [C_2_mim][OAc].

### Enzyme Activity Assay

The endoglucanase activities of Cel5A_*Tma* and its mutants were assayed at 70 °C as previously described [31]. For solid substrate assays, [C_2_mim][OAc] pretreated switchgrass (ILSG) was used instead of CMC. The enzymatic assays containing 100 µg/mL of pure enzyme and 5 % (w/v) ILSG were incubated at 70°C for 18 h. Reducing sugars were determined by DNS assay without sodium sulfite and phenol [37]. A range of D-cellobiose concentrations (0–5 mM) were used as standards for the reducing sugars. One unit of endoglucanase activity was defined as the amount of enzyme required for producing 1 μmol of cellobiose equivalents per minute.

### High Throughput Screening

To develop a robotic platform for high throughput screening of cellulase mutant libraries, the following parameters were analyzed: growth media (LB, TB, 2×YT and NZCYM) for expression, inoculation and expression methods (a two-step protocol composed of amplification and then expression and a one step protocol using direct expression), temperature and duration time for expression, protein extraction methods (BugBuster and PopCulture), DNS reagent components (with and without sulfite and phenol), sealing materials for assay plates (sealed with aluminum foil or adding light mineral oil) and incubation method (thermocycler or air-heating incubator). QFill (Genetix) was used for dispensing growth media into 96-well plates, QPix2 colony picker (Genetix) for picking mutant libraries from QMedia Trays (24.5 cm×24.5 cm, Genetix) into 96-well plates with growth media, and the Biomek FX^P^ Workstation (Beckmen Coulter) was used for protein extraction and enzyme activity assays.

**Figure 1:**
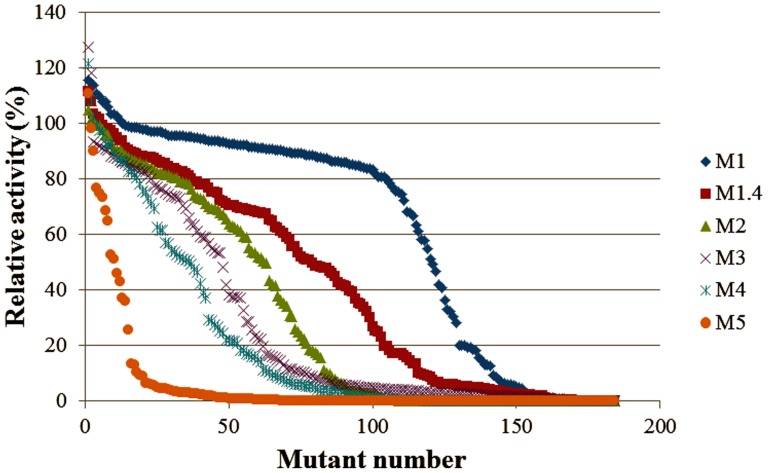
The relative activity of Cel5A_*Tma* under six different error rates was used to determine the optimal error rate for library construction. The proportion of null mutants produced using an error rate M1.4 (29 ng of *cel*5a_*Tma* gene) was ∼34 %, which indicated that the library constructed using the M1.4 error rate was suitable. The average mutation rate in the M1.4 library was ∼4.8 bp/kb gene (0.48 %)To determine the optimal error rate for Cel5A_*Tma* mutation, mutant libraries were constructed using six different error rates.

The high throughput screening protocol (Fig. 1) was as follows. Single colonies grown on QMedia Trays were picked into 96-well deep-well plates containing autoinduction medium (expression plates) using a QPix2. Expression plates were then replicated into 96-well plates containing LB with 100 ug/mL streptomycin, 0.5 % (w/v) glucose and 10 % (v/v) glycerol (archiving plates), which were then incubated 37°C for 24 h and preserved at –80°C. Expression plates were delivered to Biomek FX^P^ liquid handling system for protein extraction and enzymatic assays.

### Template preparation for random mutagenesis

The gene, *cel*5a_Tma, was amplified as described previously [31] and cloned into pET41 Ek/LIC vector (EMD Biosciences). The gene insert was confirmed by DNA sequencing (Quintarabio). The plasmid, pET41-*cel*5a_*Tma*, was extracted by QIAprep Spin Miniprep Kit (Qiagen) and the DNA concentration was determined by UV spectroscopy (NanoDrop 2000, Thermo Scientific).

### Random Mutagenesis

Primers, TmaF and TmaR (Table 1), were used for error-prone PCR amplification of the *cel*5a_*Tma* gene using a GeneMorph II Random Mutagenesis Kit (Agilent Technologies). pET41-*cel*5a_*Tma* was used as the template DNA for error-prone PCR. A 50-ul mutagenic PCR reaction contained 1x Mutazyme II reaction buffer, 200 µM of each dNTP, 1 µM of each primer, 2.5 U of Mutazyme II and a variant of template DNA (equal to 0.1–1,000 ng of *cel*5a_*Tma* gene). The mutagenic PCR were performed under the following condition - 1 cycle: 2 min at 95°C; 30 cycles: 30 s at 95°C, 30 s at 68°C, 1 min and 20 s at 72°C; 1 cycle: 10 min at 72°C. The mutagenic PCR products were gel-purified by QIAquick Gel Extraction Kit (Qiagen) and quantified by UV spectroscopy.

**Table 1:** Primers used for random and site-directed mutagenesis.

Primer	Purpose	Sequence
TmaF	RM^a^	GACGACGACAAG ATGGGGGTTGATCCGTTT
TmaR	RM^a^	GAGGAGAAGCCCGG TTATTCGATACTGTCACCGCC
L84M-F	SDM^b^	GTTATTAACGGTGCCATGAAACGCGGACTGG
K123R-F	SDM^b^	GAAACAGATTGCGGACCGTTATCGCGATTATCCGGAAACTCTGTTTTTC
N102Y-F	SDM^b^	CACCACTATGAAGAGCTGATGTATGATCCTGAAGAACATAAAG
K189Q-F	SDM^b^	CAGTTCCGAAGTGGGAGCAAAACTCCATTGTGAC
D124N-F	SDM^b^	CAGATTGCGGACCGTTATAAAAATTATCCGGAAACTCTGTTTTTC
T170A-F	SDM^b^	CCATCATTATTGGCGCCGCCGAATGGGG
R274H-F	SDM^b^	GGACAAGTTTTGTTGTACATGAAATGGAAAAGCGCCGTTG

a, random mutagenesis; b, site-directed mutagenesis. Underline letters indicate the sequence for ligation-independent cloning; Italic letters indicate the mutation nucleotides. The reverse primers not shown here are reverse and complemented to forward primers. Only forward primers were used for two or more residues replacements according to the instructions of QuikChange Lightning Multi Site-Directed Mutagenesis Kit (Agilent).

### Mutant Library Construction

Purified mutagenic fragments were cloned into pCDF2 Ek/LIC vector (EMD Biosciences) according to the manufacturer’s instructions. Mutant libraries were introduced into Acella electrocompetent cells (EdgeBio) using the Gene Pulser Xcell Electroporation System (Bio-Rad) under recommended conditions. Transformants were spread onto QMedia Trays containing LB agar media supplemented with 100 μg/mL of streptomycin and incubated at 37°C for 16–20 h until single colonies visibly formed.

### Mutant Library Preservation and Expression

To create protein expression plates, single colonies on the QMedia Trays from mutant libraries were picked into 96-well deep-well plates (2.2 mL, Thermo Scientific) using a QPix2 colony picker. These expression plates contained 0.8 mL 2×YT autoinduction media with 100 µg/mL of streptomycin [38] and were incubated in a Kuhner shaker with microtiter plate trays at 37°C for 4 h and then shifted to 30°C for an additional 22 h. After expression, these plates were replicated into archiving plates, which were 96-well shallow-well plates containing 0.15 mL LB medium with 100 μg/mL of streptomycin, 0.5 % (w/v) D-glucose and 10 % (v/v) glycerol. Archiving plates were incubated at 37°C for 24 h and stored at –80°C. In each plate, four wells were reserved as controls (three wild type inoculants as positive controls and a pCDF2 empty vector construct as a negative control). The Expression plates are then delivered to Biomek FX^P^ liquid handling system for protein extraction using PopCulture Extraction Buffer, and the resulting clear cell lysates were used for enzymatic assays.

### High Throughput Screening for Activity

A Biomek FX^P^ Workstation was used for high throughput protein extractions and enzymatic assays. PopCulture Extraction Buffer (1× rLysozyme, 1x Benzonase and 1x Proteinase Inhibitor Cocktail V EDTA-free) was dispensed into each well in expression plates, mixed by pipetting and then incubated at room temperature for 30 min. Cell debris was spun down at 4,000 rpm by 4K15 centrifuge (Sigma Laboratory Centrifuge, Germany), and the resulting supernatants were used for enzymatic assays. Supernatants were added into the reaction solution to achieve final values of 1% (w/v) CMC and 50 mM sodium citrate buffer (pH 4.80) in 96-well plates. Light mineral oil (EMD Chemicals) was used to prevent evaporation when heating. Reactions were carried out in an incubator at 70°C for 30 min. After reaction, DNS reagent was added into each well and incubated at 70°C for another 30 min. After cooling to room temperature, the assay plates were spun at 1,000 rpm and absorbance measurements were taken at 540 nm by the paradigm.

### Site-Directed Mutagenesis

Single amino acid residue substitutions were carried out using a QuikChange Lightning Site-Directed Mutagenesis Kit, while higher order (≥2) amino acid mutations were done using a QuikChange Lightning Multi Site-Directed Mutagenesis Kit according to the instructions of manufacturer (Agilent Technologies). The primers are all given in Table 1.

### Nucleotide Sequencing and Sequences Analysis

The plasmids from site-directed and random mutagenesis were amplified, extracted and the nucleotide sequences of *cel*5a_*Tma* mutants were determined by DNA sequencing. Vector NTI Advance (Life Technologies) was used to align and analyze nucleotide and amino acid sequences.

### Protein structure analysis

The coordinates of the Cel5A_*Tma* crystal structure were taken from the published structure (PDBID 3EZ8) [31] and visualized in COOT [39] and PyMOL [40]. Structures of the four single site mutants, which represent the top hits from error prone PCR (A153V, H138R, N236D and Y66F), were also generated using COOT.

### Chemicals and Reagents

All chemicals were analytical grade from Sigma-Aldrich or EMD Chemicals. pCDF-2 Ek/LIC and pET41 Ek/LIC Vector Kits, Overnight Express Autoinduction System 1, BugBuster Protein Extraction Reagent, Popculture Reagent, rLysozyme solution, Benzonase Nuclease HC (Purity >90%) and Proteinase inhibitor Cocktail V (EDTA-free) were purchased from Novagen and Calbiochem (EMD Biosciences). Luria-Bertani (LB) and Terrific Broth (TB) media were from EMD Chemicals, while 2xYT and NZCYM media from Sigma-Aldrich.

## Results

### High Throughput Screening Assay Development

A BL21 (DE3)-derived strain with double-deletions at its endA and recA alleles, Acella, eliminates the necessity for using separate cloning and expression strains, which improves the throughput for both gene cloning and library screening. Autoinduction and IPTG-induction methods yielded very similar results for expression of soluble Cel5A_*Tma* at both 20°C and 30°C. However, expression of Cel5A_*Tma* at 37°C consistently produced approximately 50 % each of “soluble-aggregated” and inactive forms (Data not shown), which may have resulted from either cell growth, or transcription and translation of recombination occurring too rapidly [41]. Therefore, a two-stage growth and induction protocol (37°C for cell growth and 30°C for protein expression) was adopted for Cel5A_*Tma* expression in 96-well plates using 2xYT autoinduction medium, which gave the best activity for Cel5A_*Tma*. The one-step protocol (direct expression) for inoculation and expression yielded similar results as the two-step procedure (amplification and then expression) and saves one day for preparing protein samples. For protein extraction, we used PopCulture instead of BugBuster reagent, because using PopCulture reagent does not require pelleting the cells, which is more suitable for processing by the liquid handling system. DNS reagent with sulfite and phenol had better sensitivity compared to the one without. However, the DNS reagent without sulfite and phenol was easier to dispose of while being sensitive enough for our screening. Adding light mineral oil into the reaction prevented evaporation both in the enzymatic reactions and DNS colorimetric reactions and gave similar results to sealing with foil. Compared to heating in thermocyclers, incubating the DNS colorimetric reactions in air-heating incubator (maximum temperature is 70°C) required a longer incubation time to yield similar color development (30 min vs. 5 min). All the steps for protein extraction, enzymatic and DNS colorimetric reactions were automated on the Biomek FX^P^ liquid handling system.

Consistent results were obtained from the cell lysates of wild-type Cel5A_*Tma* using this high throughput screening system and the coefficient of variation (CV) of this assay across three 96-well plates of WT enzyme of was less than 10 %, which is adequate for excluding false positives in most cases. Since the screening capability of this high-throughput system is 16 plates per day, ∼1,500 variants could be analyzed in one day and the defined screening job (20,000 variants) was finished within 14 days.

### Random Mutagenesis: Mutagenic Profiles of Mutazyme II for Error-prone PCR

An error rate that produces 30–40 % null mutants, defined here as having less than 10 % of the wild-type activity, corresponding to a 0.1–0.3 % mutation frequency is appropriate for library generation and screening [33]. To determine the optimal error rate for Cel5A_*Tma* mutation, mutant libraries were constructed using six different error rates where the error rate was controlled by using different amounts of template DNA. As shown in Figure 1, the proportion of null mutants produced using an error rate M1.4 (29 ng of *cel*5a_*Tma* gene) was ∼34 %, which indicated that the library constructed using the M1.4 error rate was suitable. The average mutation rate in the M1.4 library was ∼4.8 bp/kb gene (0.48 %). This mutation rate is larger than that reported to be required to achieve the same null mutant rate in directed evolution studies of mesophilic enzymes, which had a mutation frequency of 0.1–0.3 % [33], [42] but is consistent with results that showed thermophilic enzymes tend to be more stable and more mutation-tolerant than their mesophilic counterparts [43], [44].

To investigate the uniformity of mutations, the rate of introduction of frame shifts and the frequency of stop codon introduction by Mutazyme II, we sequenced a randomly chosen set of 288 variants in the M1.4 library (three 96-well plates). In total 286 kbp of sequence data was obtained and analyzed (Table 2). Similar to previously published results [45], Mutazyme II produces a uniform distribution of nucleotide transitions and transversions, which is critical for construction of random libraries. Frame shifts occur at a low frequency (deletions and insertions are lower than 3 %), and, similar to previous reports [45], the frequency of introducing stop codons was around 9 % for *cel*5a_*Tma*.

**Table 2:** Mutation spectrum of Mutazyme II in error-prone PCR of cel5a_*Tma*.

	Transversions				Transitions		Deletion	Insertion
	A↔T	G↔C	A↔C	T↔G	A↔G	T↔C		
% Total	32.02	4.82	6.14	7.02	20.18	27.19	2.19	0.44
	50				47.37			

### Primary Screen for Activity Improvement

As an initial high-throughput activity screen that was amenable to automation using robotic liquid handlers, twenty thousand variants from the M1.4 library were screened for improved total activity on carboxymethyl cellulose (CMC), which is a cellulose derivative with carboxymethyl groups (-CH2-COOH) bound to some of the hydroxyl groups of the glucopyranose monomers of the cellulose backbone and that make CMC soluble and easily dispensed into 96 well plates. E. coli cell lysates containing Cel5A_*Tma* mutants were incubated with a 1% (w/v) CMC solution at pH 4.80 and temperature 70°C for 30 min, followed by reaction with DNS reagent at 70°C for another 30 min. Of the twenty thousand mutants screened, thirty had greater than 20% higher total activity on CMC compared to wild-type Cel5A_*Tma*, and these were selected for secondary screening on CMC and on the insoluble substrate of interest, [C_2_mim][OAc]-pretreated switchgrass (ILSG).

### Secondary Screen for Specific Activity on CMC and ILSG

The thirty variants with improved total activity on CMC were expressed and purified to greater than 90% purity, and their specific activity was measured on CMC under the same reaction conditions as the *E. coli* cell lysates. Wild-type Cel5A_*Tma* had a specific activity of around 100 U/mg and fourteen of the mutants had improved specific activity. DNA sequencing revealed that twelve of these mutants were unique, while two of the fourteen were redundant variants (S177R and H138R). As shown in Table 3, the twelve unique variants have between 25 and 42% improvement in specific activity towards CMC. Nine of these are single amino acid mutants, two are double mutants (L84M-K123R and N102Y-K189Q) and one is a triple mutant (D124N-T170A-R274H).

**Table 3:** Relative specific activity of Cel5A_*Tma* improved mutants.

Clones	Relative Specific Activity (%)		Residue substitutions
	CMC	ILSG	
WT	100±3.33	100±4.38	-
P05C08	132±2.14	105±1.72	Y66F
P05D08	142±1.36	130±0.74	N236D
P06H03	126±4.01	102±3.61	L84M & K123R
P12B12 (P15H11)	125±3.71	98±3.22	S177R
G13B01 (M134B01)	130±3.41	122±4.37	H138R
G06A02	125±2.83	108±4.85	S48T
G10E12	128±3.69	92±3.90	N102Y & K189Q
G11E01	125±1.00	113±2.18	I167T
I04A12	126±4.25	111±1.76	D226E
I11E12	129±2.88	108±2.99	D124N, T170A & R274H
K12B12	128±2.47	106±3.38	W241R
N05H02	130±1.96	110±1.57	A153V

Note: CMC, carboxymethyl cellulose; ILSG, ionic liquid ([C_2_mim][OAc]) pretreated switchgrass; WT, wild type of Cel5A_*Tma*.

**Figure 2:**
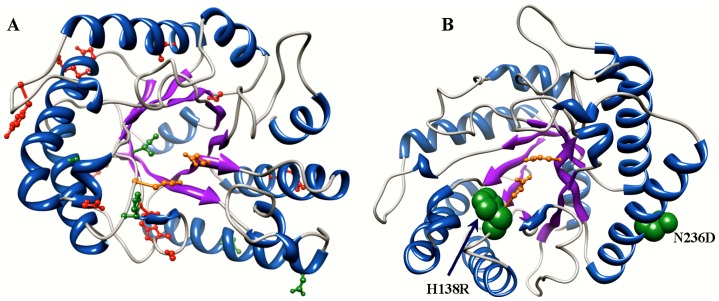
Positions of activity enhancing mutations of Cel5A_*Tma* in the crystal structure. Panel A shows the positions of mutations that yielded at least a 10% increase in specific activity on only CMC (red) and on both CMC and [C_2_mim][OAc] pretreated switchgrass (green). Panel B shows the position of the two mutations that yielded a greater than 20% increase in specific activity on [C_2_mim][OAc] pretreated switchgrass. The H138R and N236D mutations yielded a 22% and a 30% increase in specific activity on [C_2_mim][OAc] pretreated switchgrass, respectively. The double mutant (H138R + N236D) had a 33% increase in specific activity on [C_2_mim][OAc] pretreated switchgrass. In both panels the catalytic residue of Cel5A_*Tma* are colored orange.

The singular effects of the mutants having double or triple mutations were examined by assaying all possible combinations of purified single and double amino acid mutations created using site-directed mutagenesis. As shown in Table 4, their specific activities on CMC are nearly identical to either the wild type or their parent variants. Interestingly, in several cases the increase in activity on CMC of the double mutants was greater than the sum of the increases of each of the single mutants, and in some cases more than compensated for the reduced activity of one of the single mutants of the double mutant. For example, the activity on CMC of the L84M-K123R double mutant was 26% higher than the wild-type activity, while the L84M single mutant activity was only 97.5% that of the WT, and the L123R mutant was only 20% more active than the WT. Similarly, the N102Y-K189Q double mutant has 28% greater activity than the wild-type, while the N102Y mutant and the K189Q single mutants had only a 6% increase and a 10% increase, separately. As highlighted in Figure 2A, Structurally, these synergistic mutations are far apart and distal to the active Cel5A_*Tma* active site and validate that subtle nonlinear interactions in proteins play a critical role in optimal enzyme function.

**Table 4:** Relative specific activities of Cel5A_*Tma* variants with different combinations of mutations.

Parent Clone	Relative Specific Activity (%)		Amino Acid Substitutions
	CMC	ILSG	
WT	100±3.33	100±4.38	-
P06H03	97±5.56	92±2.14	L84M
	120±4.12	96±2.07	K123R
G10E12	106±3.85	100±2.32	N102Y
	110±1.20	90±4.21	K189Q
I11E12	120±1.94	104±3.77	D14N
	95±2.87	98±2.64	T170A
	110±3.61	99±2.98	R274H
	122±3.08	107±2.45	D124N + T170A
	120±2.57	101±3.17	D124N + R274H
	105±4.35	106±1.07	T170A + R274H
P05D08 + G13B01	140±1.19	133±4.16	N236D + H138R

Note: CMC, carboxymethyl cellulose; ILSG, ionic liquid treated switchgrass; WT, wild type of Cel5A_Tma.

Given that CMC is a soluble substrate with different physical characteristics (e.g., molecular weight, solubility) than the insoluble amorphous form of cellulose produced from pretreatment of switchgrass with [C2mim][OAc] ([46], [47] and that cellulose derivatized with carboxymethyl groups may have a different mode of binding to Cel5A_*Tma* compared to cellulose from ILSG, the expectation that improved activity on CMC will directly translate to improved activity on ILSG is uncertain. To verify the activity improvement on a biorefinery relevant feedstock, the twelve mutants with improved activity on CMC were screened, using the DNS assay, on ILSG.

As shown in Table 3, two single mutation variants showed increased specific activity on ILSG: variant N236D had a 30% increase and variant H138R had a 22% increase. These improvements are due to improved activity of Cel5A_*Tma* on the cellulose component of ILSG. While Cel5A_*Tma* has both cellulase and mannanase activities [32], switchgrass contains essentially no mannan (∼0.3%) [48]–[50]. So the increases in reducing ends measured by the DNS assay, which measures reducing ends from any sugar polymer present, are due to increased activity on cellulose. Based on structural analyses, a plausible explanation for the enhanced activity of the H138R mutant is that it stabilizes the position of the catalytic E136 residue and the structure of the reaction intermediate (Figure 2B). As shown in Figure 2B, the N236D is on the surface of Cel5A_*Tma* distal to the active site. A possible explanation of the improved activity of this mutant is that mutation of the polar asparagine residue to the charged aspartate residue stabilizes the protein structures due to a solvent screening effect. Thus the two mutants that increase Cel5A_*Tma* activity on both CMC and ILSG appear to do so through orthogonal mechanisms. Since the variants with mutations, N236D and H138R, showed the largest increase in activity on ILSG, a double mutant (N236D + H138R) consisting of the combination these two mutations was constructed and assayed for activity on both CMC and ILSG. The effect of these two mutations is not additive; compared to the most active single mutant parent the double mutant has similar activities towards both CMC (40% increase) and ILSG (33% increase) (Table 4).

The structure of Cel5A_*Tma* exhibits a single-domain architecture composed exclusively of a catalytic domain [31]. Cel5A_*Tma* belongs to family 5 of glycoside hydrolases and members of this group show a common (β/α)_8_ TIM-barrel fold. To gain a better understanding of the relationship between improved activity and structural variation for the Cel5A_*Tma* mutants with improved activity, we analyzed the position of the mutations using the crystal structure of Cel5A_*Tma* [31]. The twelve improved activity Cel5A_*Tma* variants exhibit 16 mutation sites, because some of the variants have double or triple mutations (Table 3). As shown in Figure 2A, ten of the sixteen (62.5%) mutations from the twelve variants are located in the loop regions, while the other six are in the secondary structural elements, and, with the exception of mutants A153V and I167T, fourteen of the sixteen mutations are at surface residues. Interestingly, the mutations that resulted in the greatest increases in activity are distal to the active site, demonstrating that activity enhancing mutations need not be limited to the active site and suggesting One possible explanation is that the distal mutations increased the thermostability of Cel5A_*Tma*, resulting in an apparent increase in activity due to an increase in the time the enzyme can catalyze cellulose hydrolysis at 70 °C. However, the reaction time of these assays was 30 min, which is much shorter than the half life of Cel5A_*Tma* (20 hours at 80°C [19]), so increased thermostability won’t affect specific activities measured at 70°C. Other possible explanations are that these types of distal mutations increase specific activity by enhancing the coordination of dynamic modes associated with activity or by biasing the folding landscape toward the most active conformational state, resulting in a larger number of optimally folded structures in the conformational ensemble.

## Discussion

Improving the efficiency of the saccharification process for the production of fuels from lignocellulosic biomass can be achieved by enhancing the catalytic efficiency and stability of lignocellulolytic enzymes. Directed evolution of thermophilic cellulases for increased activity has typically met many obstacles, owing primarily to the lack of efficient high throughput screening platforms or selection systems for thermophilic enzymes [25]. Here, we developed an integrated and efficient high-throughput robotics platform for screening the activity of large libraries of thermophilic cellulases generated by random mutagenesis. The platform is flexible and easily adaptable to screen large libraries of glycoside hydrolases from, for example, metagenomic or directed evolution experiments whose activity assays are based on the production of reducing sugars from soluble or insoluble polysaccharides.

We used this high-throughput screening platform to screen a library of mutants of a thermophilic endoglucanase, Cel5A_*Tma*, for improved specific activity on a biorefinery relevant substrate, namely [C_2_mim][OAc] pretreated switchgrass. We generated a library of 20,000 variants using an optimal average mutation rate of ∼4.8 bp/kb and screened them first on CMC followed by screening of the most promising mutants on [C_2_mim][OAc] pretreated switchgrass. In the first screen of the Cel5A_*Tma* mutant library, twelve variants were confirmed to have 25–42% improvement specific activity on CMC. To test whether these variants have higher specific activity against solid substrates, we assayed their activities on [C_2_mim][OAc] pretreated switchgrass. Compared to the wild type, most of these mutants except N236D and H138R showed little improvement on [C_2_mim][OAc]-pretreated switchgrass. Two mutants, N236D and H138R, showed a significant increase in specific activity on ILSG, although the magnitude is smaller than that towards CMC (30% vs. 42% for N236D; 22% vs. 30% for H138R). These results demonstrate the necessity to screen enzymes for activity on the particular substrate for which their activity is being engineered.

The activity enhancing mutations that are distal to the active demonstrate the complexities of engineering enhanced activity into a group of enzymes (GH5 family) with highly homologous active sites and that have evolved primarily to maintain their stability and activity in different environments such as higher temperatures and high salt concentrations. In this context, the absence of activity enhancing mutations in the active site is not surprising and it raises the possibility that the enhancement in activity observed here is due to effects on the dynamics of the active site that either lead to a more optimal preorganization of the catalytic residues in the transition state [51], [52] or that lead to more optimized dynamics that are associated with the catalytic step [53]–[55]. Alternatively, the presence of mutations on the surface of the protein may change the way the protein interacts with the solvent system present, which may result in enhanced folding kinetics or biasing the equilibrium toward the folded state with highest activity, resulting in a larger number of protein molecules in the ensemble being folded in the enzymatically most favorable state (i.e., folding is biased toward a larger fraction of enzymes are folded properly) [56], [57]. Rational designs based on mutations distal to the active site that are calculated to alter active site dynamics or to bias the folding funnel to the most favorable fold have, to our knowledge, not been reported and are likely outside the realm of expectations for structure guided design, necessitating the development and use of high-throughput approaches to screening large libraries of randomly generated mutants.

Ionic liquids are a promising new approach to biomass pretreatment and, compared to other pretreatment methods such as dilute acid and AFEX, have been shown to produce higher total glucose yields and faster rates of glucose production [13]. However, the use of ionic liquids presents downstream challenges for the enzymes that have to remain active in their presence. Here, using a random mutagenesis combined with a new high-throughput screening protocol for thermophilic enzymes, we have generated two mutants (N236D and H138R) of Cel5A_*Tma* that have increased activity on [C_2_mim][OAc] pretreated switchgrass relative to wild-type Cel5A_*Tma*. The ability to improve the activity of cellulases on IL pretreated biomass will allow for the optimization of enzyme cocktails for this environment, which in turn will reduce the cost of enzymes by improving the kinetics of saccharification.
